# A case of cutaneous angiosarcoma with intradermal metastases responding to PD-1 inhibition-based immunotherapy

**DOI:** 10.1016/j.jdcr.2025.05.046

**Published:** 2025-07-09

**Authors:** Kayla Danesh, Jenny C. Hu, Niels Kokot, Zachary Goldstein, Brittney DeClerck, Bryce Beutler, Hossein Jadvar, Katherine Butcher, Gino K. In

**Affiliations:** a Department of Dermatology, Keck School of Medicine, University of Southern California, Los Angeles, California; b Department of Otolaryngology, Keck School of Medicine, University of Southern California, Los Angeles, California; c Department of Pathology, Keck School of Medicine, University of Southern California, Los Angeles, California; d Department of Radiology, Keck School of Medicine, University of Southern California, Los Angeles, California; e Division of Oncology, Norris Comprehensive Cancer Center, University of Southern California, Los Angeles, California

**Keywords:** angiosarcoma, anti–PD-1, FDG PET/CT, immune checkpoint inhibitor therapy, low TMB, PD-L1 negative, pembrolizumab, pseudoprogression

## Introduction

Cutaneous angiosarcoma is an aggressive and rare malignant tumor with a poor prognosis. The most common location for this tumor is the head and neck region, and patients may present with multiple lesions, some of which may resemble bruises or hemangiomas.^1^ Because of extensive skin involvement, surgical resection with negative margins may be challenging.^1^ Current treatment options for unresectable or metastatic disease include chemotherapy with taxanes or anthracyclines, although these options may have both limited efficacy and significant toxicity.^2^ Immune checkpoint inhibitors targeting programmed death-1 (PD-1) or programmed death-ligand 1 (PD-L1) present a promising new treatment option for cutaneous angiosarcoma, which may potentially be more effective and also well tolerated.^3^

## Case report

**Fig 1:**
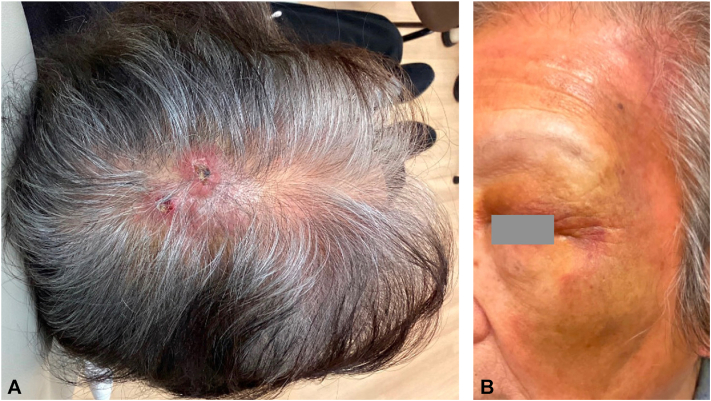
Physical examination. **A,** Two hyperkeratotic nodules on the mid-crown of the anterior and posterior scalp. **B,** Faint purpura, erythema, yellowing, and swelling of the forehead, periorbital area, and left cheek.

**Fig 2:**
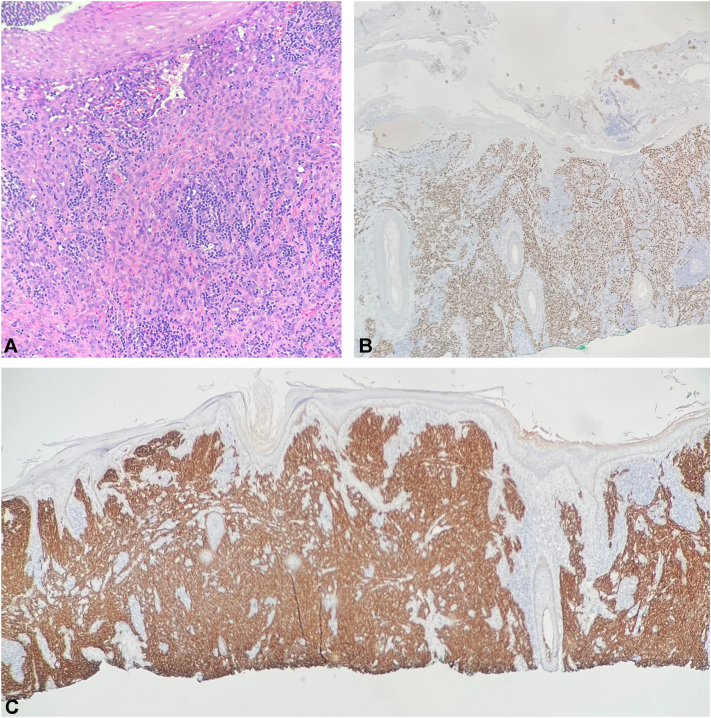
Biopsy specimens from the scalp. **A,** Hematoxylin-eosin staining showing dense, diffuse atypical epithelioid appearing proliferation throughout the dermis with somewhat hyperchromatic nuclear detail and abundant cytoplasm. Focally, irregular vascular channels dissecting between dense collagenous bundles are appreciated as well as scattered mononuclear cells. **B,** Significant staining of lesional cells of interest appreciated with ERG staining. **C,** Significant staining of lesional cells of interest appreciated with D2-40 staining. (**A,** Hematoxylin-eosin stain; original magnifications: **A,** ×20).

A 90-year-old Asian woman with hypertension and diabetes presented with a several-month history of a raised scalp lesion. Examination revealed 2 hyperkeratotic nodules on the midcrown of the anterior and posterior scalp, coalescing into each other (Fig 1, *A*). There was faint purpura, erythema, yellowing, and swelling of the bilateral forehead, left periorbital area, and left cheek (Fig 1, *B*). Shave biopsies of this raised scalp lesion showed dense, diffuse, atypical epithelioid appearing proliferation throughout the dermis with somewhat hyperchromatic nuclear detail and abundant cytoplasm (Fig 2, *A*). Focally, irregular vascular channels dissecting in between dense collagenous bundles were appreciated as well as scattered mononuclear cells (Fig 2, *A*). Immunohistochemistry was performed for further evaluation and confirmation of the diagnosis, including SOX10, p40, Desmin, SMA, CD10, ERG, D2-40, and cMYC (with significant staining of lesional cells of interest appreciated with ERG and D2-40) (Fig 2, *B* and *C*). These findings confirmed a diagnosis of cutaneous angiosarcoma.

**Fig 3:**
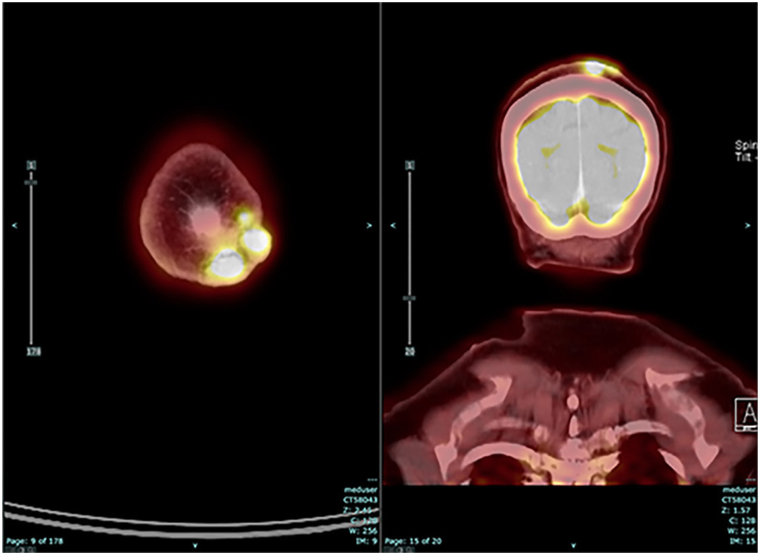
Fused 18F-fluorodeoxyglucose (FDG)-PET-CT axial and coronal images before therapy, size of scalp lesion 2.9 cm × 4.0 cm, SUV_max_ 13. *FDG*, fluorodeoxyglucose; *PET-CT*, positron emission tomography-computed tomography; *SUV*_*max*_, maximum standardized uptake value.

**Fig 4:**
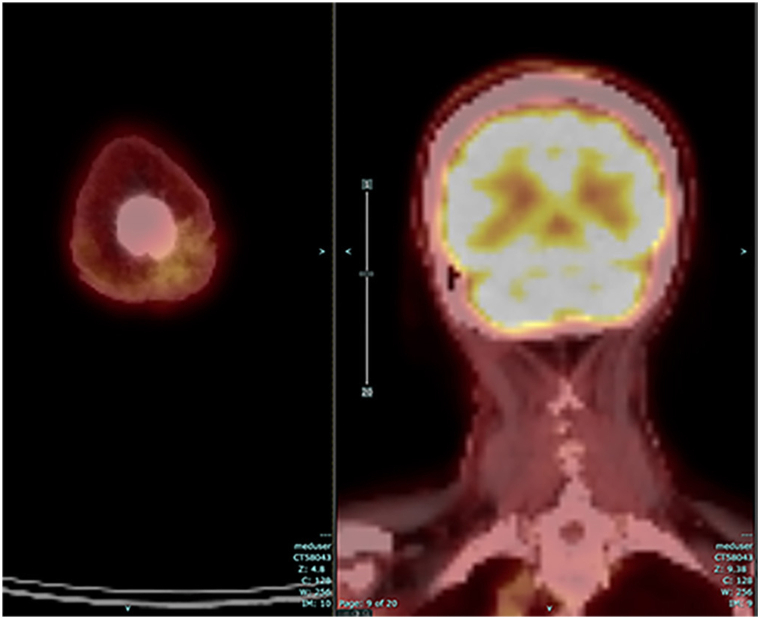
Fused FDG PET-CT axial and coronal images after 5 cycles of anti–PD-1 therapy; size of scalp lesion decreased from 2.9 cm × 4.0 cm to 2.2 × 3.6 cm (and less dense), and SUV_max_ decreased from 13 to 2.6, compatible with a favorable response to treatment. *FDG*, fluorodeoxyglucose; *PD-1*, programmed death-1; *PET-CT*, positron emission tomography-computed tomography; *SUV*_*max*_, maximum standardized uptake value.

Positron emission tomography-computed tomography (PET-CT) with 18F-flourodeoxyglucose (FDG) showed a hypermetabolic conglomerate of soft tissue nodules and masses centered within the left vertex scalp, approximately 2.9 × 4.0 cm in size, with maximum standardized uptake value (SUV_max_) 13, involving the dermal and subdermal tissues and abutting the underlying outer table of the calvarium, with no evidence of metastatic disease (Fig 3). The patient was initially planned for surgical resection but rapidly developed multiple new subcutaneous nodules on the right side of her face and left temple during the subsequent weeks. Punch biopsies confirmed metastatic angiosarcoma; she was referred to medical oncology for systemic therapy. Given her cutaneous metastasis without visceral involvement, as well as her age (years) and functional status, PD-1 monotherapy with pembrolizumab 200 mg every 3 weeks was recommended. After 2 cycles, she continued to develop new subcutaneous lesions along the right forehead; given the absence of side effects and the possibility of pseudoprogression, as defined by immune Response Evaluation Criteria in Solid Tumors,^4^ her treatment continued. After the third cycle, significant improvement was noted, with resolution of the forehead or scalp lesions. Repeat FDG PET-CT scan after the fifth cycle showed only residual, nonmeasurable nodularity and low metabolic activity with SUV_max_ 2.6, compatible with favorable treatment response, and no evidence of metastatic disease (Fig 4). Mutational analysis by next-generation sequencing of the tumor revealed a tumor mutational burden (TMB) of 8 mutations or Mb, and immunohistochemistry PD-L1 expression (clone used: SP142) was negative (0%). The patient was continued on pembrolizumab, but the dosing was switched to 400 mg every 6 weeks. After the sixth cycle, the patient developed a mild rash with macules and papules, which was managed with topical hydrocortisone, and low-grade arthralgias that were managed conservatively. CT scans after the seventh and ninth cycles showed no evidence of progression, and the patient also showed clinical signs of response. The patient has been on therapy for 10 months and recently completed the tenth cycle of therapy; she continues to have the same mild adverse effects, with a sustained response to treatment and no other complications.

## Discussion

Here, we present a case of an elderly patient with a PD-L1-negative, low TMB scalp angiosarcoma who had a complete response to PD-1 inhibition-based immunotherapy, highlighting the potential use of this therapy in this rare cutaneous malignancy. This case report is in line with a growing number of individual reports and retrospective studies that demonstrate that cutaneous angiosarcoma is responsive to anti–PD-1 therapy. However, this case demonstrates a clear response to anti–PD-1 therapy in a patient with both a PD-L1-negative and a low TMB cutaneous angiosarcoma. There have been reports of angiosarcoma patients with both “intermediate” or “high” TMB responding to immune checkpoint inhibitor therapy^5^^,^^6^; our patient had a TMB of 8 mutations or Mb, but cutoffs for the definitions of these “intermediate” or “high” ranges vary from one study to another.^5^^,^^6^ Furthermore, large genomic studies suggest that cutoffs for response to PD-1 therapy may differ by tumor type,^7^ and this needs to be further investigated in cutaneous angiosarcoma specifically. PD-L1 expression is also a controversial biomarker for responsiveness to PD-1 blockade, given that patients with PD-L1 negative tumors have still been reported to exhibit a significant response to anti–PD-1 therapy,^8^ as in the case of our patient. Clinically, some studies have suggested that cutaneous angiosarcomas of the head or neck are particularly responsive to PD-1,^3^ presumably because of increased UV radiation and associated mutations and tumor neoantigens. Because our case report demonstrates that neither PD-L1-positive expression nor high TMB is required for a response to PD-1 inhibition-based immunotherapy, we advocate that patients with cutaneous angiosarcoma be considered for this therapy, given limited alternatives.

To our knowledge, this is also the first report of a patient with cutaneous angiosarcoma experiencing pseudoprogression when treated with PD-1 inhibition. Multiple studies have described pseudoprogression, the development of enlarging or new tumor lesions followed by subsequent evident treatment response, in patients with metastatic solid tumors after initiation of anti–PD-1 therapy.^4^ Although the mechanism for this phenomenon is not entirely understood, it is thought that it may be related to a late response to therapy or to an influx of immune cells and subsequent tumor necrosis after initiation of therapy.^4^^,^^9^

Importantly, we highlight the utility of FDG PET or CT not only to verify the eventual response to anti–PD-1 therapy but also particularly so after initial pseudoprogression. Prior studies have described PET or CT in determining clinical outcomes for cutaneous angiosarcoma.^10^ Meanwhile, there is also a growing role for PET or CT in determining response to therapy for patients with solid tumors treated with anti-PD-1.^11^ Further investigation is needed to clarify the relevant predictive biomarkers to identify which patients will respond to anti–PD-1 therapy for this rare tumor type.

In conclusion, we present a unique case of cutaneous angiosarcoma that was responsive to PD-1 therapy in leading to a complete response despite multiple cutaneous metastases, setting the stage for further investigation into the use of this cancer immunotherapy for similar patients.

## Conflicts of interest

None disclosed.

## References

[1] Patel S.H., Hayden R.E., Hinni M.L. (2015). Angiosarcoma of the scalp and face: the Mayo Clinic experience. JAMA Otolaryngol Head Neck Surg.

[2] Sindhu S., Gimber L.H., Cranmer L., McBride A., Kraft A.S. (2017). Angiosarcoma treated successfully with anti-PD-1 therapy—a case report. J Immunother Cancer.

[3] Hamacher R., Kämpfe D., Reuter-Jessen K. (2018). Dramatic response of a PD-L1-positive advanced angiosarcoma of the scalp to pembrolizumab. JCO Precis Oncol.

[4] Mönch S., Heimer M.M., Winkelmann M. (2023). Patterns of pseudoprogression across different cancer entities treated with immune checkpoint inhibitors. Cancer Imaging.

[5] Amagai R., Fujimura T., Kambayashi Y. (2023). Recurrent, tumor mutation burden-high, cutaneous angiosarcoma of the scalp treated with pembrolizumab. Case Rep Oncol.

[6] Florou V., Rosenberg A.E., Wieder E. (2019). Angiosarcoma patients treated with immune checkpoint inhibitors: a case series of seven patients from a single institution. J Immunother Cancer.

[7] Samstein R.M., Lee C.H., Shoushtari A.N. (2019). Tumor mutational load predicts survival after immunotherapy across multiple cancer types. Nat Genet.

[8] Grossman J.E., Vasudevan D., Joyce C.E., Hildago M. (2021). Is PD-L1 a consistent biomarker for anti-PD-1 therapy? The model of balstilimab in a virally-driven tumor. Oncogene.

[9] Jia W., Gao Q., Han A., Zhu H., Yu J. (2019). The potential mechanism, recognition and clinical significance of tumor pseudoprogression after immunotherapy. Cancer Biol Med.

[10] Vasanawala M.S., Wang Y., Quon A., Gambhir S.S. (2006). F-18 fluorodeoxyglucose PET/CT as an imaging tool for staging and restaging cutaneous angiosarcoma of the scalp. Clin Nucl Med.

[11] Donegani M.I., Ferrarazzo G., Marra S. (2020). Positron emission tomography-based response to target and immunotherapies in oncology. Medicina (Kaunas).

